# Human biodistribution and dosimetry of [^11^C]-UCB-J, a PET radiotracer for imaging synaptic density

**DOI:** 10.1186/s40658-021-00384-5

**Published:** 2021-04-23

**Authors:** Christopher Cawthorne, Paul Maguire, Joel Mercier, David Sciberras, Kim Serdons, Guy Bormans, Jan de Hoon, Koen Van Laere, Michel Koole

**Affiliations:** 1 Nuclear Medicine and Molecular Imaging, Department of Imaging and Pathology, KU Leuven, Leuven, Belgium; 2 UCB Pharma, Braine l’Alleud, Belgium; 3 Division of Nuclear Medicine, University Hospitals Leuven, Leuven, Belgium; 4 Laboratory for Radiopharmaceutical Research, KU Leuven, Leuven, Belgium; 5 Center for Clinical Pharmacology, University Hospitals Leuven, Leuven, Belgium

**Keywords:** [^11^C]-UCB-J, Synaptic density, Radiation dosimetry, Human biodistribution, OLINDA

## Abstract

**Rationale:**

[^11^C]-UCB-J is an emerging tool for the noninvasive measurement of synaptic vesicle density in vivo. Here, we report human biodistribution and dosimetry estimates derived from sequential whole-body PET using two versions of the OLINDA dosimetry program.

**Methods:**

Sequential whole-body PET scans were performed in 3 healthy subjects for 2 h after injection of 254 ± 77 MBq [^11^C]-UCB-J. Volumes of interest were drawn over relevant source organs to generate time-activity curves and calculate time-integrated activity coefficients, with effective dose coefficients calculated using OLINDA 2.1 and compared to values derived from OLINDA 1.1 and those recently reported in the literature.

**Results:**

[^11^C]-UCB-J administration was safe and showed mixed renal and hepatobiliary clearance, with largest organ absorbed dose coefficients for the urinary bladder wall and small intestine (21.7 and 23.5 μGy/MBq, respectively). The average (±SD) effective dose coefficient was 5.4 ± 0.7 and 5.1 ± 0.8 μSv/MBq for OLINDA versions 1.1 and 2.1 respectively. Doses were lower than previously reported in the literature using either software version.

**Conclusions:**

A single IV administration of 370 MBq [^11^C]-UCB-J corresponds to an effective dose of less than 2.0 mSv, enabling multiple PET examinations to be carried out in the same subject.

**Trial registration:**

EudraCT number: 2016-001190-32. Registered 16 March 2016, no URL available for phase 1 trials.

## Introduction

Synaptic vesicle glycoprotein 2A (SV2A) is an integral presynaptic vesicle membrane protein and is expressed in presynaptic vesicles throughout the brain [1–3]. Reduction in synaptic vesicle density measured ex vivo post-mortem has been reported in numerous neurological pathologies including Alzheimer’s disease, Parkinson’s disease, Huntingdon’s disease, Down syndrome, major depression, stroke, and epilepsy. In addition, in epilepsy, SV2A has been confirmed as the target of the anti-epileptic drugs levetiracetam and brivaracetam [4, 5]. Noninvasive measurement of synaptic density has the potential to allow early detection of disease and improved prognosis, as well as enabling measurement of target engagement in early clinical drug development of agents based on the levetiracetam pharmacophore [6]. Primate studies showed [^11^C]-UCB-J to be an excellent tracer with good pharmacokinetic properties and it is currently the compound most frequently used for a variety of neurophysiological investigations (reviewed in [7]). The distribution volume (V_T_) of [^11^C]-UCB-J has been correlated with both SV2A and synaptophysin expression level in the primate brain [1]. Subcortical white matter has been validated as a reference tissue to facilitate quantitative clinical studies [8, 9]. To support clinical translation of this tracer and in addition to a similar recent human biodistribution study by the Yale PET group [10], we report here human biodistribution and dosimetry estimates for [^11^C]-UCB-J derived using OLINDA/EXM version 1.1 (to allow comparison with the previous report). In addition, we also derived and compared human dosimetry estimates using the more recent and commercially available version of OLINDA/EXM (version 2.1), as this makes use of voxel-based computational phantoms (with organ masses scaled to match the computational phantoms reported in ICRP 89), updated tissue weighting factors from ICRP103, and the more recent human alimentary tract model [11] and so should provide the best current estimate of human dosimetry.

## Materials and methods

### Subjects

Three healthy volunteers were included (2 females and 1 male, 35.3 ± 11.8 years, 74.7 ± 15.0 kg), free of current medical or psychiatric illnesses as determined by medical history, laboratory findings, and clinical examination. Patient demographics are summarized in Table 1. Vital signs were monitored before injection of [^11^C]-UCB-J, at 5, 10, 20, and 45 minutes (min) post injection and at the end of the final scan.

**Table 1:** Demographic information, injected dose, and injected mass

Patient	Sex	Age	Weight	Injected activity (MBq)	Injected mass (μg)	Injected mass (μg/kg)
1	F	29	57.4	337.33	4.15	0.072
2	F	28	85.0	238.47	3.22	0.038
3	M	49	81.6	186.18	2.24	0.027

This study was part of a larger phase I, single-center, open-label study (EudraCT Number 2016-001190-32). Written informed consent was obtained for each subject. The study was approved by the local ethical committee and conducted in accordance with the most recent version of the Declaration of Helsinki.

### Radioligand synthesis

[^11^C]-UCB-J radiosynthesis was carried out under full GMP as previously described [9]. The radioligand was obtained with high radiochemical purity (>99%) and molar activity (25.5±1.5 GBq/μmol) (at the time of injection, average value from 3 batches).

### Biodistribution and whole-body dosimetry

For each subject, PET data were obtained over approximately 2 h after IV bolus injection of 254± 77 MBq (range 186 to 337 MBq) of [^11^C]-UCB-J. The mass dose for UCB-J was 3.20 ± 0.96 μg (range 2.24 to 4.15 μg). Individual injected activities and mass doses are also summarized in Table 1. PET data were acquired on a Siemens Biograph 16 PET/CT camera (Siemens, Erlangen, Germany) in two segments with the field of view covering from the head to the upper thigh. The first segment (sequential WB scans 1 to 8) began concurrently with the start of injection and lasted for approximately 60 min (time per bed position was 30 s [for WB 1–3], 60 s [for WB 4–6], and 120 s [for WB 7 and 8]). The second segment (WB 9) started at 1.25 h post-injection with 4 min per bed position. A low-dose CT (11 mAs) was performed before each scan segment for both PET attenuation correction and to provide anatomical information. There was no excretion by any subject during the scan period.

## Data analysis

### Dosimetry

Whole-body PET scans were reconstructed using the ordered subset expectation maximization (OSEM) algorithm (5 iterations, 8 subsets, Gaussian postfilter of 6.0 mm FWHM, zoom 1.4) using the manufacturer’s software. Corrections for randoms, scatter, and attenuation (via low-dose CT) were included in each reconstruction. Three-dimensional volumes of interest (VOIs) representing entire source organs were manually delineated on PET images, with each co-registered CT scan used to verify anatomical location. Eleven organs were selected on the basis of significant and visually assessable tracer uptake over the entire acquisition: brain, gallbladder (2 out of 3 subjects), small intestine, stomach, heart wall, kidneys, liver, lungs, red marrow, spleen, and urinary bladder. Large bone structures (large vertebrae, pelvis) were delineated based on visible uptake and considered as a surrogate for red marrow. Activity in the whole body was also calculated in order to quantify activity uptake outside of the selected organs, to be entered as “other” or “remainder” in the dose calculation software.

Time-activity curves were obtained for each source organ by calculating the non-decay corrected total activity in the volumes of interest expressed as a percentage of the total injected dose, using PMOD (version 3.9, PMOD Technologies LLC, Zurich, Switzerland). Where significant tracer was observed in the injection line, this was quantified via delineation of a VOI and subtracted from the injected dose. Time-integrated activity coefficients (i.e., normalized cumulated activities (NCAs) or “residence times”) for [^11^C]-UCB-J were calculated as the area under the time-activity curves of each source organ through curve fitting with the most appropriate model [12]. A multi-exponential curve model A × (1−exp(−B × T)) × exp(−C × T) + D × exp(−E × T) was used to fit brain, stomach, heart wall, and liver uptake while a bi-exponential curve model A × exp(−B × T) + C × exp(−D × T) was used for red marrow and spleen uptake. In addition, a trapezoid model was used for the gallbladder, kidneys, and urinary bladder uptake, while uptake in the lungs were fitted with A × exp(−B × T) and the remainder with A × (1−exp(−B × T)) + C × exp(−D × T). For all curve models, T represented the time post tracer injection.

Absorbed dose coefficients were calculated using the Organ Level Internal Dose Assessment (OLINDA/EXM) software package versions 1.1 (Vanderbilt University, USA) and 2.1 (Hermes Medical Solutions, Stockholm, Sweden). The average fraction of activity entering the intestinal VOI was used as an input to determine NCAs for the components of the gastrointestinal tract using the International Commission on Radiological Protection (ICRP) 30 Gastrointestinal Tract model [13] or ICRP 100 human alimentary tract (HAT) model [14] as implemented in OLINDA/EXM versions 1.1 and 2.1 respectively.

For OLINDA/EXM version 1.1, a sex-matched model was applied to calculate effective dose coefficient values to ICRP60. For OLINDA/EXM version 2.1, sex-averaged effective dose coefficient values to ICRP 103 were calculated by entering NCAs for each organ into the male and female phantoms respectively. For both versions, gastrointestinal tract values were derived with the GI and HAT models as described above, and overall effective dose coefficients were derived by averaging the average male and female values derived above.

## Results

### Adverse events

There were no adverse events in any of the 3 subjects after injection of [^11^C]-UCB-J. No significant changes in vital signs (i.e., pulse rate, blood pressure, respiratory rate) or electrocardiograms were observed.

### Biodistribution and dosimetry

**Fig. 1:**
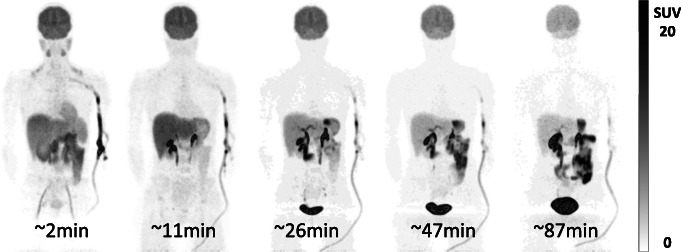
Maximum intensity projection PET images of subject AN01 showing distribution of radioactivity in whole-body (head to thigh) after injection of 337 MBq of [^11^C]-UCB-J. High radioactivity uptake is visible in the brain

**Fig. 2:**
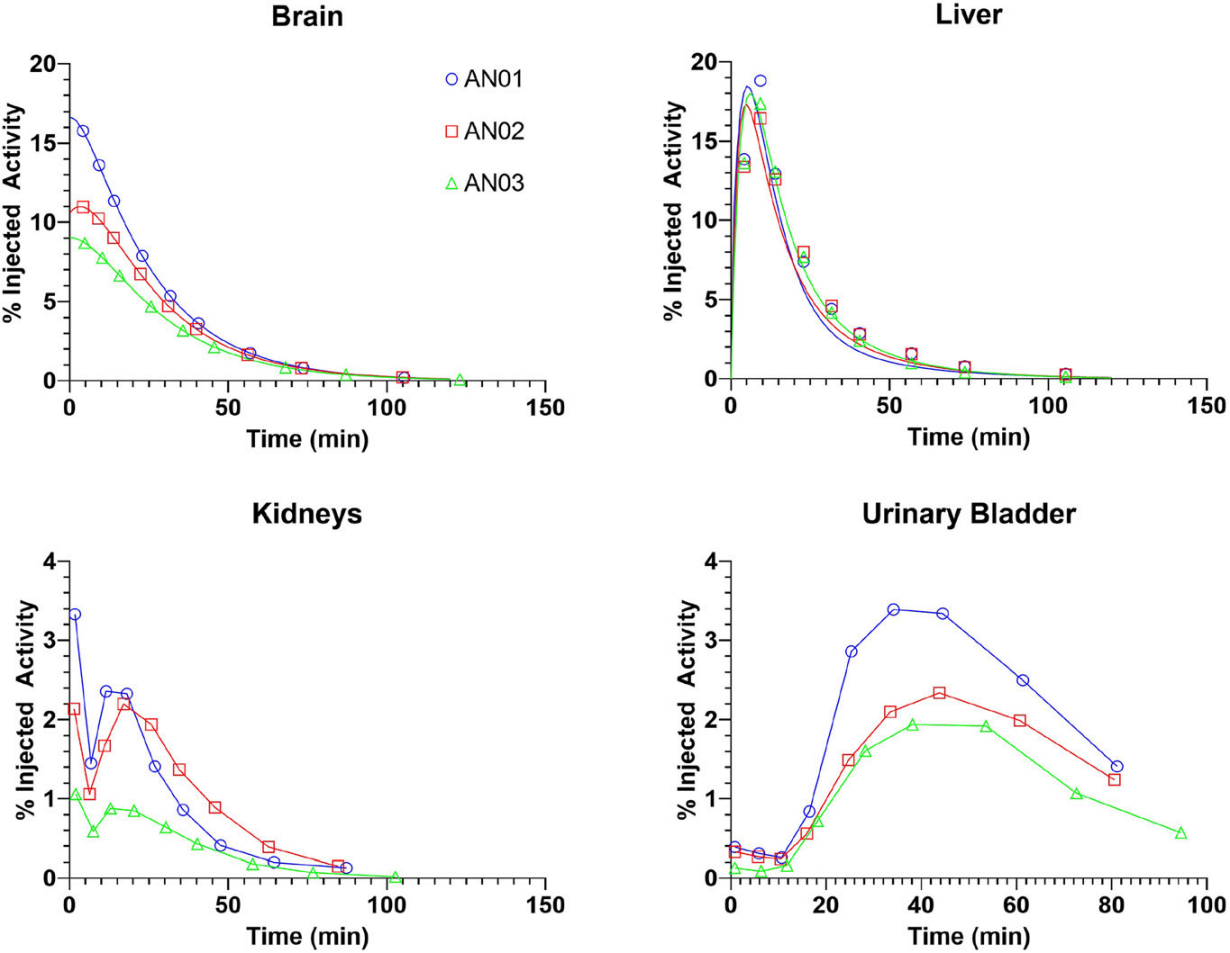
Time-activity curves of select source organs (brain [**a**], liver [**b**], kidneys [**c**], and urinary bladder [**d**]) after [^11^C]-UCB-J injection in 3 subjects. Graphs indicate non-decay corrected mean organ radioactivity over time, expressed as % injected activity (open symbols). Points were directly connected for trapezoid approximation (**c**, **d**) or were fitted by exponential curves (**a**, **b**)

Visual inspection of the sequential WB images showed high early uptake in both the brain and liver, with the kidney, urinary bladder, and gastrointestinal uptake consistent with a mixture of renal and hepatobiliary clearance of intact and metabolized tracer (Fig. 1). Time-activity curves for the brain, liver, kidneys, and urinary bladder are shown in Fig. 2. The highest initial uptake of radioactivity was found in the liver, with peak values ranging from 17 to 19% of injected activity followed by subsequent clearance over the duration of the scan. Kidney and urinary bladder activity peaks at 20 and 40 min, respectively, indicating early renal clearance, while increasing activity in the gastrointestinal tract indicates later hepatobiliary clearance of the tracer.

Normalized cumulated activities (NCAs) are given for all source organs in Table 2. The liver demonstrated the highest exposure (0.667 ± 0.003 MBq-hr/hr), while the lowest was the lower large intestine (0.0002 ± 0.0001 MBq-hr/hr).

**Table 2:** Normalized cumulated activity coefficients (NCAs) for indicated source organs determined from whole-body imaging of three healthy subjects injected intravenously with [^11^C]-UCB-J

Source organ	NCA (MBq-hr/hr)			Average
	Adult female	Adult female	Adult male	
Brain	0.0787	0.0633	0.0506	0.0642 ± 0.0141
Gallbladder	0.0030	0.0000	0.0014	0.0015 ± 0.0015
Small intestine	0.0524	0.0341	0.0598	0.0488 ± 0.0133
Right colon	0.0062	0.0041	0.0071	0.0058 ± 0.0015
Left colon	0.0002	0.0001	0.0003	0.0002 ± 0.0001
Stomach	0.0105	0.0047	0.0078	0.0077 ± 0.0029
Heart wall	0.0037	0.0034	0.0053	0.0041 ± 0.0010
Kidneys	0.0144	0.0156	0.0064	0.0121 ± 0.0050
Liver	0.0654	0.0641	0.0705	0.0667 ± 0.0034
Lungs	0.0101	0.0093	0.0121	0.0105 ± 0.0014
Red marrow	0.0211	0.0194	0.0192	0.0199 ± 0.0010
Spleen	0.0015	0.0017	0.0008	0.0013 ± 0.0005
Urinary bladder	0.0358	0.0263	0.0211	0.0277 ± 0.0075
Total body/remainder	0.1720	0.1946	0.1872	0.1846 ± 0.0155

Using OLINDA/EXM 1.1, organ absorbed dose coefficients were largest for the urinary bladder wall (23.3 μSv/MBq), small intestine (18.6 μSv/MBq), brain (15.3 μSv/MBq), liver (14.1 μSv/MBq), and kidneys (11.8 μSv/MBq) (Table 3). Differences between organ absorbed dose coefficients from this study and those previously reported are also presented in Table 3. The overall effective dose coefficient (mean ± SD) was 5.4 ± 0.7 μSv/MBq. Using OLINDA/EXM 2.1, organ absorbed dose coefficients were largest for the urinary bladder wall (21.7 μSv/MBq), small intestine (23.5 μSv/MBq), brain (14.4 μSv/MBq), liver (14.6 μSv/MBq), and kidneys (11.9 μSv/MBq) (Table 4). The effective dose coefficient (mean ± SD) was 5.1 ± 0.8 μSv/MBq. Differences between calculated absorbed dose coefficient for OLINDA/EXM 1.1 and 2.1 are also presented in Table 4.

**Table 3:** Absorbed dose coefficients for [^11^C]-UCB-J determined from three healthy subjects using OLINDA/EXM 1.1 compared to literature values

Target organ	Absorbed dose coefficients (mSv/MBq)					
	Adult female	Adult female	Adult male	Average	Average [10]	% Difference
Adrenals	3.26E−03	3.28E−03	2.57E−03	2.92E−03 ± 0.50	3.34E−03	−12%
Brain	2.09E−02	1.69E−02	1.17E−02	15.30E−03 ± 5.09	1.85E−02	−17%
Breasts	1.56E−03	1.66E−03	1.32E−03	1.47E−03 ± 0.21	1.75E−03	−16%
Gallbladder wall	1.20E−02	4.14E−03	6.95E−03	7.51E−03 ± 0.79	1.22E−02	−38%
LLI wall	3.62E−03	3.13E−03	2.95E−03	3.16E−03 ± 0.30	2.20E−02	−86%
Small intestine	2.08E−02	1.43E−02	1.97E−02	18.63E−03 ± 1.52	2.83E−03	558%
Stomach wall	8.82E−03	5.27E−03	6.16E−03	6.60E−03 ± 0.63	6.83E−03	−3%
ULI wall	8.55E−03	6.35E−03	7.96E−03	7.71E−03 ± 0.36	2.83E−03	173%
Heart wall	5.53E−03	5.19E−03	5.62E−03	5.49E−03 ± 0.18	4.44E−03	24%
Kidneys	1.57E−02	1.67E−02	7.29E−03	11.75E−03 ± 6.30	1.01E−02	16%
Liver	1.56E−02	1.52E−02	1.27E−02	14.05E−02 ± 1.91	1.98E−02	−29%
Lungs	4.50E−03	4.25E−03	4.09E−03	4.23E−03 ± 0.20	5.24E−03	−19%
Muscle	2.09E−03	2.09E−03	1.70E−03	1.90E−03 ± 0.28	2.11E−03	−10%
Ovaries	4.05E−03	3.46E−03	3.31E−03	3.53E−03 ± 0.32	3.50E−03	1%
Pancreas	3.43E−03	3.23E−03	2.72E−03	3.03E−03 ± 0.43	3.37E−03	−10%
Red marrow	4.21E−03	3.96E−03	3.77E−03	3.93E−03 ± 0.22	2.22E−03	77%
Osteogenic cells	4.69E−03	4.63E−03	3.42E−03	4.04E−03 ± 0.88	3.11E−03	30%
Skin	1.53E−03	1.59E−03	1.25E−03	1.41E−03 ± 0.22	1.64E−03	−14%
Spleen	4.35E−03	4.57E−03	2.40E−03	3.43E−03 ± 1.46	4.60E−03	−25%
Testes			1.43E−03	1.43E−03	2.16E−03	−34%
Thymus	1.82E−03	1.95E−03	1.55E−03	1.72E−03 ± 0.24	2.06E−03	−16%
Thyroid	1.59E−03	1.71E−03	1.49E−03	1.57E−03 ± 0.11	1.87E−03	−16%
Urinary bladder wall	3.50E−02	2.62E−02	1.59E−02	23.25E−02 ± 4.84	2.21E−02	5%
Uterus	4.43E−03	3.76E−03	3.41E−03	3.75E−03 ± 0.48	3.21E−03	17%
**Total body**	**3.33E**−**03**	**3.15E**−**03**	**2.56E**−**03**	2.90E−03 ± 0.48	3.12E−03	−7%
**Effective dose coefficient (mSv/MBq) (ICRP 60)**	**6.53E**−**03**	**5.35E**−**03**	**4.91E**−**03**	5.43E−03 ± 0.73	7.59E−03	−28%

**Table 4:** Absorbed dose coefficients for [^11^C]-UCB-J determined from three healthy subjects using OLINDA/EXM 2.1, compared to OLINDA/EXM 1.1

Target organ	Absorbed dose coefficients (mSv/MBq)				
	Adult female	Adult female	Adult male	Average	% Difference
Adrenals	4.99E−03	4.84E−03	4.13E−03	4.52E−03 ± 0.56	55%
Brain	1.93E−02	1.56E−02	1.13E−02	14.38E−02± 4.35	−6%
Breasts	1.56E−03	1.65E−03		1.61E−03 ± 0.06	10%
Esophagus	2.63E−03	2.62E−03	2.36E−03	2.49E−03 ± 0.19	n/a
Eyes	2.93E−03	2.73E−03	1.87E−03	2.35E−03 ± 0.68	n/a
Gallbladder wall	1.28E−02	4.19E−03	7.95E−03	8.22E−03 ± 0.39	9%
Left colon	3.62E−03	3.18E−03	3.82E−03	3.61E−03 ± 0.30	14%
Small intestine	2.67E−02	1.82E−02	2.46E−02	23.53E−02 ± 1.52	26%
Stomach wall	9.32E−03	5.58E−03	6.76E−03	7.11E−03 ± 0.49	8%
Right colon	8.63E−03	6.52E−03	9.06E−03	8.32E−03 ± 1.05	8%
Rectum	3.96E−03	3.48E−03	2.62E−03	3.17E−03 ± 0.78	n/a
Heart wall	5.33E−03	4.97E−03	5.82E−03	5.49E−03 ± 0.47	0%
Kidneys	1.60E−02	1.68E−02	7.33E−03	11.87E−02 ± 6.41	1%
Liver	1.58E−02	1.54E−02	1.35E−02	14.55E−02 ± 1.49	4%
Lungs	4.09E−03	3.85E−03	3.80E−03	3.89E−03 ± 0.12	−8%
Ovaries	3.33E−03	3.02E−03		3.18E−03 ± 0.22	68%
Pancreas	4.66E−03	4.16E−03	3.89E−03	4.15E−03 ± 0.37	17%
Prostate			2.50E−03	2.50	n/a
Salivary glands	2.64E−03	2.52E−03	2.00E−03	2.29E−03 ± 0. 41	n/a
Red marrow	4.81E−03	4.55E−03	3.67E−03	4.18E−03 ± 0.71	6%
Osteogenic cells	3.40E−03	3.23E−03	2.88E−03	3.10E−03 ± 0.31	−23%
Spleen	5.23E−03	5.34E−03	2.80E−03	4.04E−03 ± 1.76	18%
Testes	0.00E+00	0.00E+00	1.38E−03	1.38E−03	−3%
Thymus	2.10E−03	2.17E−03	1.86E−03	2.00E−03 ± 0.19	16%
Thyroid	1.74E−03	1.83E−03	1.57E−03	1.68E−03 ± 0.15	7%
Urinary bladder wall	3.12E−02	2.34E−02	1.61E−02	21.70E−02 ± 7.92	−7%
Uterus	4.56E−03	3.88E−03	0.00E+00	4.22E−03 ± 0.48	12%
**Total body**	3.45E−03	3.20E−03	2.39E−03	2.86E−03 ± 0.66	−1%
**Effective dose coefficient (mSv/MBq) (ICRP 103)**	6.24E−03	5.07E−03	4.50E−03	5.08E−03 ± 0.82	−6%

## Discussion

Imaging synaptic density has investigated with a number of PET ligands targeting the SV2A presynaptic vesicle glycoprotein with [^11^C]-UCB-J having the best pharmacological characteristics [7] and being the most established clinically to date [1, 8, 9, 15–27]. This study reports radiation dosimetry for [^11^C]-UCB-J using two versions of the OLINDA dosimetry software. As previously reported, [^11^C]-UCB-J was well tolerated in all healthy volunteers at the doses administered for PET scanning.

**Fig. 3:**
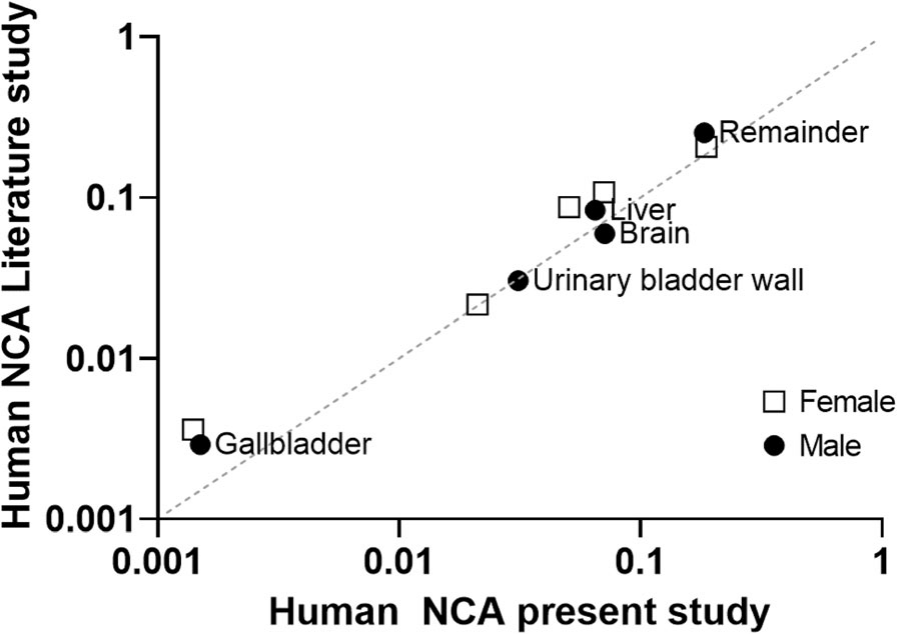
Log-log plot of human organ residence times for the current study vs those reported previously [10]

NCAs were in general lower in the current study compared to those previously reported [10], though the urinary bladder wall, brain, liver, and kidneys were similarly identified as among those receiving the highest radiation exposure. These data and comparison to [10] are also summarized in Table 2 and Fig. 3. Uptake in the gastrointestinal tract was considerably different; however, though this may to some extent reflect the different methodologies employed (direct segmentation vs. use of the ICRP GI tract models to give regional NCAs); as the gallbladder was not discernible in one patient in the current study, this also lowered the overall average for the current work. With the exception of the GI tract, organs with maximal uptake calculated using OLINDA 1.1 were identical to the previous report; however, individual organ absorbed dose coefficient estimates identified the urinary bladder wall as the critical organ in both sexes (as is the case for about 30% of [^11^C]-labeled radiotracers [28]); absorbed dose coefficient to liver was markedly lower in females. Overall, these variations may be attributed to individual differences between patients both in organ size and shapes and radiotracer clearance coupled with the small sample sizes used in both studies (and only one male in the current study; see Table 1).

Although overall effective dose coefficients were lower than those previously reported, they were still higher than dosimetry estimates from preclinical studies in non-human primates (3.3-3.4μSv/MBq ED) [29], it should be noted that this may reflect simple scaling based on subject weight, which does not account for the difference in the size of organs relative to overall weight between species [30, 31]. The largest differences were seen in the stomach, urinary bladder wall, and gallbladder as previously reported [10]. Although the molar activity in our study was lower than previously reported in our center and others [8–10], the injected mass is still predicted to give <1% occupancy based on the predicted in vivo affinity of UCB-J [29] and is below the amount specified for use in clinical studies [32].

When comparing results between OLINDA/EXM version 1.1 vs 2.1, individual organ doses were generally higher (74% of comparable organs) using the more recent version; however, the same organs with highest organ doses were identified and overall effective dose coefficient was lower (5.1 ± 0.8 vs 5.4 ± 0.7 μSv/MBq). Differences in organ doses can be attributed to the use of non-uniform rational B-spline (NURBS) phantoms scaled to match the organ masses given in ICRP 89 [33], rather than the “mathematical phantoms” based on simpler geometric shapes developed by Cristy-Eckerman that were used in OLINDA 1.1. In addition, the human alimentary tract (HAT) model defined in ICRP 100 was used for OLINDA 2.1, superseding the previous model based on ICRP 30 [13] used in OLINDA 1.1; differences in overall effective dose coefficient will reflect these changes as well as the use of the different tissue weighting factors from ICRP 103 rather than ICRP 60 (tissue weighting factors decrease for both liver and bladder), as has been reported in previous studies [34, 35].

The effective dose coefficient for [^11^C]-UCB-J was 5.4 ± 0.7 μSv/MBq using OLINDA/EXM version 1.1; however, values from both OLINDA versions were comparable with other reported effective dose coefficients for C-11 labeled PET tracers [28, 36]. In Europe, the maximum allowable dose for most studies using radiopharmaceuticals for young healthy volunteers (below 50 years) is 10 mSv per year (WHO Class IIB, where benefit for medical knowledge is targeted) [37], equating to 1969 MBq of [^11^C]-UCB-J (estimated using OLINDA/EXM 2.1). CT would thus be the largest contributor to radiation dose for [^11^C]-UCB-J PET/CT.

## Conclusion

Clinical use of [^11^C]-UCB-J is safe and results in an ED of 5.1 ± 0.8 μSv/MBq, confirming previous findings and allowing multiple serial PET scanning to be performed in patients without exceeding the annual dose limitations. The use of different versions of OLINDA resulted in relatively minor differences in calculated effective dose coefficient.

## Data Availability

Data generated as part of this study are available from the corresponding authors on reasonable request.

## References

[1] Finnema SJ, Nabulsi NB, Eid T, Detyniecki K, Lin SF, Chen MK (2016). Imaging synaptic density in the living human brain. Sci Transl Med.

[2] Bajjalieh SM, Frantz GD, Weimann JM, McConnell SK, Scheller RH (1994). Differential expression of synaptic vesicle protein 2 (SV2) isoforms. J Neurosci.

[3] Bajjalieh SM, Peterson K, Shinghal R, Scheller RH (1992). SV2, a brain synaptic vesicle protein homologous to bacterial transporters. Science..

[4] Lynch BA, Lambeng N, Nocka K, Kensel-Hammes P, Bajjalieh SM, Matagne A (2004). The synaptic vesicle protein SV2A is the binding site for the antiepileptic drug levetiracetam. Proc Natl Acad Sci U S A.

[5] Nowack A, Malarkey EB, Yao J, Bleckert A, Hill J, Bajjalieh SM (2011). Levetiracetam reverses synaptic deficits produced by overexpression of SV2A. PLoS One.

[6] Mercier J, Provins L, Valade A (2017). Discovery and development of SV2A PET tracers: potential for imaging synaptic density and clinical applications. Drug Discov Today Technol.

[7] Cai Z, Li S, Matuskey D, Nabulsi N, Huang Y (2019). PET imaging of synaptic density: a new tool for investigation of neuropsychiatric diseases. Neurosci Lett.

[8] Finnema SJ, Nabulsi NB, Mercier J, Lin SF, Chen MK, Matuskey D (2018). Kinetic evaluation and test-retest reproducibility of [(11)C]UCB-J, a novel radioligand for positron emission tomography imaging of synaptic vesicle glycoprotein 2A in humans. J Cereb Blood Flow Metab.

[9] Koole M, van Aalst J, Devrome M, Mertens N, Serdons K, Lacroix B (2019). Quantifying SV2A density and drug occupancy in the human brain using [(11)C]UCB-J PET imaging and subcortical white matter as reference tissue. Eur J Nucl Med Mol Imaging.

[10] Bini J, Holden D, Fontaine K, Mulnix T, Lu Y, Matuskey D (2020). Human adult and adolescent biodistribution and dosimetry of the synaptic vesicle glycoprotein 2A radioligand (11)C-UCB-J. EJNMMI Res.

[11] Stabin MG, Siegel JA (2018). RADAR dose estimate report: a compendium of radiopharmaceutical dose estimates based on OLINDA/EXM version 2.0. J Nucl Med.

[12] Bolch WE, Eckerman KF, Sgouros G, Thomas SR (2009). MIRD pamphlet no. 21: a generalized schema for radiopharmaceutical dosimetry--standardization of nomenclature. J Nucl Med.

[13] ICRP (1979). Publication 30, part 1, 1979. Ann ICRP.

[14] International Commission on Radiological P (2006). Human alimentary tract model for radiological protection. ICRP publication 100. A report of the international commission on radiological protection. Ann ICRP.

[15] Chen MK, Mecca AP, Naganawa M, Finnema SJ, Toyonaga T, Lin SF (2018). Assessing synaptic density in Alzheimer disease with synaptic vesicle glycoprotein 2A positron emission tomographic imaging. JAMA Neurol.

[16] Finnema SJ, Rossano S, Naganawa M, Henry S, Gao H, Pracitto R (2019). A single-center, open-label positron emission tomography study to evaluate brivaracetam and levetiracetam synaptic vesicle glycoprotein 2A binding in healthy volunteers. Epilepsia..

[17] Finnema SJ, Toyonaga T, Detyniecki K, Chen MK, Dias M, Wang Q (2020). Reduced synaptic vesicle protein 2A binding in temporal lobe epilepsy: a [(11) C]UCB-J positron emission tomography study. Epilepsia..

[18] Holmes SE, Scheinost D, Finnema SJ, Naganawa M, Davis MT, DellaGioia N (2019). Lower synaptic density is associated with depression severity and network alterations. Nat Commun.

[19] Mansur A, Rabiner EA, Comley RA, Lewis Y, Middleton LT, Huiban M (2020). Characterization of 3 PET tracers for quantification of mitochondrial and synaptic function in healthy human brain: (18)F-BCPP-EF, (11)C-SA-4503, and (11)C-UCB-J. J Nucl Med.

[20] Matuskey D, Tinaz S, Wilcox KC, Naganawa M, Toyonaga T, Dias M (2020). Synaptic changes in Parkinson disease assessed with in vivo imaging. Ann Neurol.

[21] Mecca AP, Chen MK, O’Dell RS, Naganawa M, Toyonaga T, Godek TA (2020). In vivo measurement of widespread synaptic loss in Alzheimer’s disease with SV2A PET. Alzheimers Dement.

[22] Mertens N, Maguire RP, Serdons K, Lacroix B, Mercier J, Sciberras D (2020). Validation of parametric methods for [(11)C]UCB-J PET imaging using subcortical white matter as reference tissue. Mol Imaging Biol.

[23] Naganawa M, Gallezot JD, Finnema S, Matuskey D, Mecca AP, Nabulsi NB, et al. Simplified quantification of (11)C-UCB-J PET evaluated in a large human cohort. J Nucl Med. 2021;62(3):418–21. 10.2967/jnumed.120.243949 PMC8049341 32646875

[24] Nicastro N, Holland N, Savulich G, Carter SF, Mak E, Hong YT (2020). (11)C-UCB-J synaptic PET and multimodal imaging in dementia with Lewy bodies. Eur J Hybrid Imaging.

[25] O’Dell RS, Mecca AP, Chen MK, Naganawa M, Toyonaga T, Lu Y (2021). Association of Abeta deposition and regional synaptic density in early Alzheimer’s disease: a PET imaging study with [(11)C]UCB-J. Alzheimers Res Ther.

[26] Rossano S, Toyonaga T, Finnema SJ, Naganawa M, Lu Y, Nabulsi N (2020). Assessment of a white matter reference region for (11)C-UCB-J PET quantification. J Cereb Blood Flow Metab.

[27] Smart K, Liu H, Matuskey D, Chen MK, Torres K, Nabulsi N, et al. Binding of the synaptic vesicle radiotracer [(11)C]UCB-J is unchanged during functional brain activation using a visual stimulation task. J Cereb Blood Flow Metab. 2021;41(5):1067–79. 10.1177/0271678X20946198 PMC8054713 32757741

[28] van der Aart J, Hallett WA, Rabiner EA, Passchier J, Comley RA (2012). Radiation dose estimates for carbon-11-labelled PET tracers. Nucl Med Biol.

[29] Nabulsi NB, Mercier J, Holden D, Carre S, Najafzadeh S, Vandergeten MC (2016). Synthesis and preclinical evaluation of ^11^C-UCB-J as a PET tracer for imaging the synaptic vesicle glycoprotein 2A in the brain. J Nucl Med.

[30] Hall C, Lueshen E, Mosat A, Linninger AA (2012). Interspecies scaling in pharmacokinetics: a novel whole-body physiologically based modeling framework to discover drug biodistribution mechanisms in vivo. J Pharm Sci.

[31] Petrulli JR, Hansen SB, Abourbeh G, Yaqub M, Bahce I, Holden D (2017). A multi species evaluation of the radiation dosimetry of [(11)C]erlotinib, the radiolabeled analog of a clinically utilized tyrosine kinase inhibitor. Nucl Med Biol.

[32] Milicevic Sephton S, Miklovicz T, Russell JJ, Doke A, Li L, Boros I (2020). Automated radiosynthesis of [(11) C]UCB-J for imaging synaptic density by positron emission tomography. J Labelled Comp Radiopharm.

[33] Stabin MG, Siegel JA, Xu XG (2018). SNMMI RADARCot. RADAR develops new generation of dosimetry phantoms. J Nucl Med.

[34] Gnesin S, Cicone F, Mitsakis P, Van der Gucht A, Baechler S, Miralbell R (2018). First in-human radiation dosimetry of the gastrin-releasing peptide (GRP) receptor antagonist (68)Ga-NODAGA-MJ9. EJNMMI Res.

[35] Melendez-Alafort L, Ferro-Flores G, De Nardo L, Bello M, Paiusco M, Negri A (2019). Internal radiation dose assessment of radiopharmaceuticals prepared with cyclotron-produced (99m) Tc. Med Phys.

[36] Zanotti-Fregonara P, Innis RB (2012). Suggested pathway to assess radiation safety of ^11^C-labeled PET tracers for first-in-human studies. Eur J Nucl Med Mol Imaging.

[37] Radiation protection 99: Guidance on medical exposures in medical and biomedical research 1998 [cited 2020 24/11/2020]. Available from: https://ec.europa.eu/energy/sites/ener/files/documents/099_en.pdf.

